# A new intelligent bearing fault diagnosis model based on triplet network and SVM

**DOI:** 10.1038/s41598-022-08956-w

**Published:** 2022-03-28

**Authors:** Kaisi Yang, Lianyu Zhao, Chenglin Wang

**Affiliations:** 1 School of Computer Science and Engineering, Tianjin University of Technology, Tianjin, 300384 China; 2 Tianjin Key Laboratory of Advanced Mechatronic System Design and Intelligent Control, School of Mechanical Engineering, Tianjin University of Technology, Tianjin, 300384 China

**Keywords:** Mechanical engineering, Computer science

## Abstract

Separating sensitive characteristic signals from original vibration data is an important challenge for rolling bearing fault diagnosis. Because it is difficult to obtain large number of damaged bearings, Rolling bearing fault datasets are often small sample datasets. For the classification of small sample rolling bearing fault datasets, we propose a coupling vibration data classification method based on triplet embedding. The method is divided into two steps: feature extraction and fault identification. First, build a triple embedding based on the CNN model to reduce the original vibration signal, and then train the SVM model for classification. Compared with traditional features and autoencoder, triplet network can learn the differences between samples. Make classification training easier and more accurate. We have evaluated the performance of this method through two bearing experiment examples. The experimental results show that this method is superior to stacked autoencoder, stacked denoising autoencoder and CNN.

## Introduction

### Research status of bearing fault diagnosis

Bearing is a typical component of rotating machinery. The quality of bearing directly determines the performance of rotating machinery. Accurate and timely fault detection is the key technology to ensure the reliability and safety of bearings. Due to the wide application of bearing in various industries, the maintenance cost of bearing fault is high, the consequences of bearing fault are serious. So the fault diagnosis of bearing and condition monitoring are very active research fields, which is very helpful for the early warning and fault location of rotating machinery. At present, on-line diagnosis and prediction of machine condition are used for fault early warning and maintenance.

With the progress of sensor technology and signal processing technology, we can obtain higher precision vibration signal, acoustic signal, current signal, voltage signal, temperature signal and so on. In recent years, the research of bearing fault diagnosis is gradually increasing, and the research based on Shaw learning is in-depth^1–8^. In order to solve over decomposition and the problem of information loss, Li et al.^9^ have proposed Independence-oriented VMD to identify wheelset bearing faults orderly. For early fault prediction of bearings, Li et al.^10^ have proposed adaptive multi-scale morphological analysis and bandwidth empirical mode decomposition. Zhang et al.^11^ have proposed A bearing fault diagnosis method using variational mode decomposition. Through the analysis of the failure mechanism, they established the fault signal calculation model of the defects in different positions of the rolling bearing. In order to realize the adaptive separation of Fourier spectrum, Zheng et al.^12^ have proposed the adaptive parameterless EWT method. Wu et al.^13^ have used the fully integrated empirical mode decomposition of adaptive noise and the Hilbert-Huang transform method to extract multiple degraded features in the degraded feature extraction stage. They selected monotonous, robust, and fault-related degradation characteristics, and merged them with the Mahalanobis distance health index as the main component. Yan et al.^14^ have proposed a fault classification algorithm based on SVM optimized by multiple features. The fault feature information is extracted from time domain by VMD, FFT and statistical analysis, frequency domain and time-frequency domain respectively.

There are also a lot of researches on bearing fault diagnosis models based on deep learning^15–23^. Xia et al.^24^ have proposed a multi-sensor-based CNN model, and classification accuracy of the model on the Case Western Reserve University bearing dataset reached 99.41%. Liu et al.^25^ have proposed a dislocated time series CNN model, which uses the dislocated time series of the original signal to train CNN. Jiang et al.^26^ have proposed a multi-scale CNN, and the classification accuracy on the Case Western Reserve bearing dataset reached 98.53%. Zhang et al.^27^ have proposed a long short memory recurrent neural network model to evaluate the bearing performance degradation. Zhao et al.^28^ have developed a variant of the deep residual network model, which uses dynamic weighted wavelet coefficients to optimize the deep residual network to improve the diagnostic performance. The input of the network is a series of wavelet packet coefficient sets in different frequency bands. Wang et al.^29^ have proposed a method to transform the vibration information of multiple sensors into image information. This method can integrate the information and obtain more abundant features than single sensor vibration signal. Yan et al. has done a lot of research in this field, The models they studied including a novel architecture named multiscale cascading deep belief network(MCDBN) for identify the fault location of the rotating machinery; a novel approach called multi-domain indicator-based optimized stacked denoising autoencoder for automatic fault identification of rolling bearing and a novel hybrid deep learning model for multistep forecasting of diurnal wind speed^30–32^.

There are also a lot of researches on transfer learning and small sample model prediction^33–37^.

### Problems of existing models

1. Traditional feature extraction methods, such as empirical mode decomposition, wavelet transform, fast Fourier transform, etc. need a lot of expert experience. Because of the sensor noise, interference, shafting misalignment, etc. the initial fault is easy to be covered by clutter, difficult to show in the time-frequency spectrum. This makes it difficult to find the initial bearing fault;
2. The end-to-end network has strong nonlinear fitting capabilities, but requires a large amount of sample data. Different from the image classification task, the test conditions of the industrial bearing dataset are harsh. It requires high-precision vibration sensors, servo motors, a set of high-precision shafting, and a stable data acquisition and control system. And it is difficult to obtain a large number of samples of damaged bearings. The public datasets are generally small sample datasets. However, the advantages of end-to-end networks on small sample datasets are not obvious, and it is difficult to learn the distribution of samples;
3. Widely used autoencoder networks(AE) such as stacked autoencoders(SAE), stacked denoising autoencoders(SDAE), etc. can map data to lower dimensions. Then use traditional classification algorithms, such as SVM, random forest, etc. to classify, and you can get better results on small sample datasets. But the prime objective of the autoencoder is to encode data into low-dimensional expression, and then restore the low-dimensional expression to the original signal as much as possible. Only pay attention to whether the restored signal is slightly different from the original signal, and some important information used for classification may be discarded as noise, which makes the classification results of algorithms such as SVM and random forest inaccurate;

## Fault diagnosis using the triplet network and SVM

This paper mainly studies the problem of bearing fault diagnosis. These damaged parts include the inner ring of the bearing, the balls, and the outer ring of the bearing. Use the vibration sensor to collect the vibration of the shaft system and analyze the vibration signal changes caused by the damage of different parts of the bearing. Because the bearing will produce a certain amount of vibration during high-speed rotation due to shaft deflection, machining error, etc., it is difficult to identify early fault signals. Moreover, because the bearing failure dataset is generally a small sample dataset, it is difficult to obtain lots of bearing data of different parts of the failure. This further challenges the possibility of using a single feature in bearing fault classification.

### Basic SAE and SDAE

AE is a kind of artificial neural network. AE updates network weights through unsupervised learning to learn the mapping of input data to feature space. AE consists of an encoder and a decoder. The encoder *f* maps the input data to a low dimensional space through function $h = f\left( x \right)$. To obtain a low dimensional representation *h* of the original data. The decoder *g* maps *h* back to the input space through the function ${\hat{x}} = g\left( h \right)$ to obtain the reconstructed data ${\hat{x}}$ of *x*. Generally, the loss function of a network can be defined as:

1 $$\begin{aligned} L = \frac{1}{n}\sum \limits _{i = 1}^n {\left\| {{x_i} - Net({x_i})} \right\| } \end{aligned}$$

**Figure 1:**
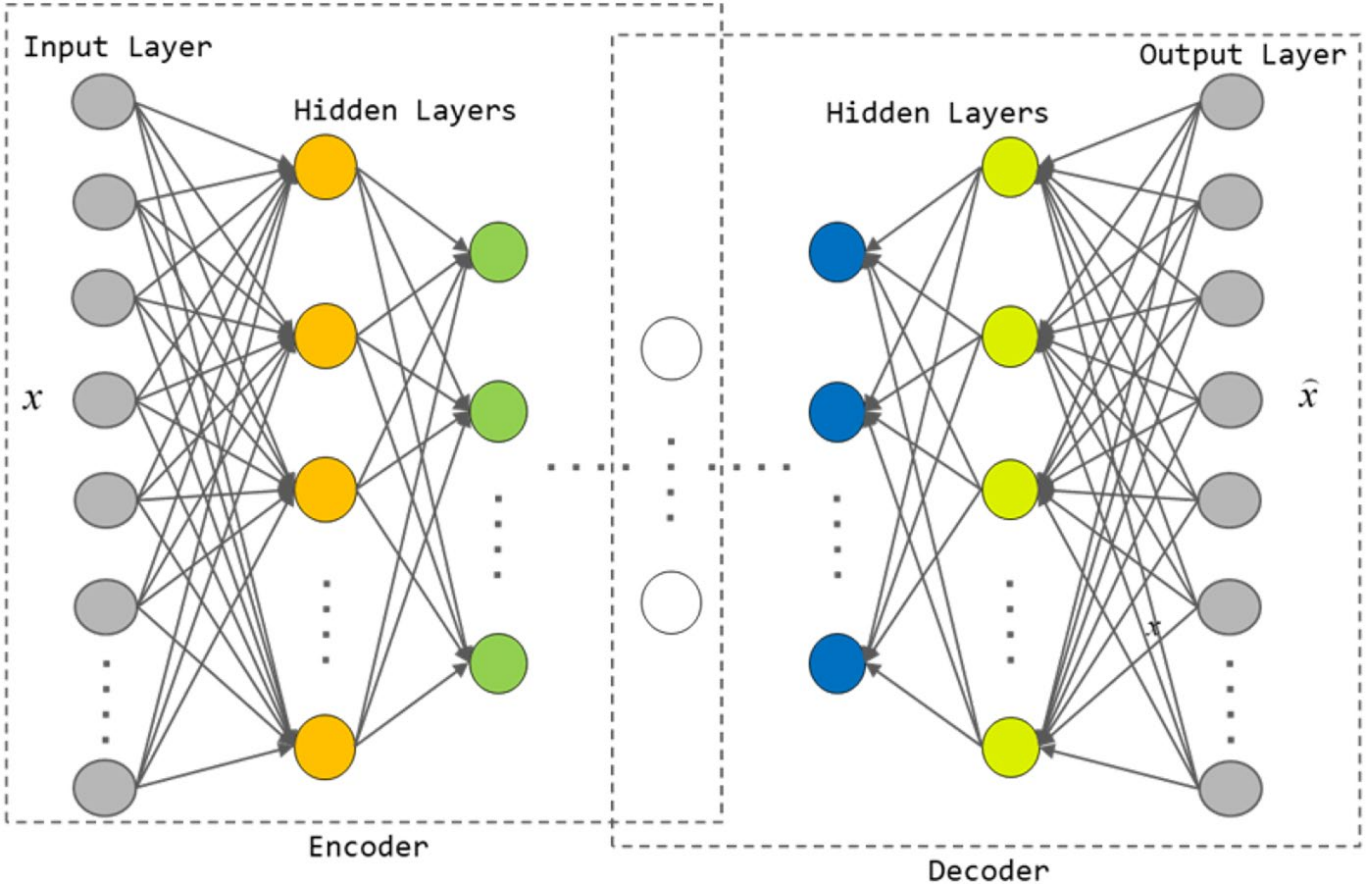
Hierarchical framework of SAE.

The loss function calculates the Euclidean distance between *x* and ${\hat{x}}$ to reduce the reconstruction error and obtain the reconstructed data closer to the original data. The output *h* of the encoder can be used as a low dimensional representation of the input data. The encoder and decoder of SAE are composed of multiple AE nested to form a structure with multiple hidden layers. Compared with AE, SAE can better learn the deep features of the original data.The structure of SAE is shown in Fig. 1.

The basic idea of DAE is to reconstruct the original input from a damaged input to obtain a robust representation of the original input. This can prevent AE from learning only the mapping between input and construction output, and capture more informative hidden patterns. In general, the input *x* is added to the noise $\sigma$ to get the damage signal ${\tilde{x}}$ form *x*, and then ${\tilde{x}}$ is input to the DAE to get the reconstructed data of ${\hat{x}}$, Formula 2 calculates the loss function:

2 $$\begin{aligned} L = \frac{1}{n}\sum \limits _{i = 1}^n {\left\| {{x_i} - Net\left( {{x_i} + {\sigma _i}} \right) } \right\| } \end{aligned}$$

**Figure 2:**
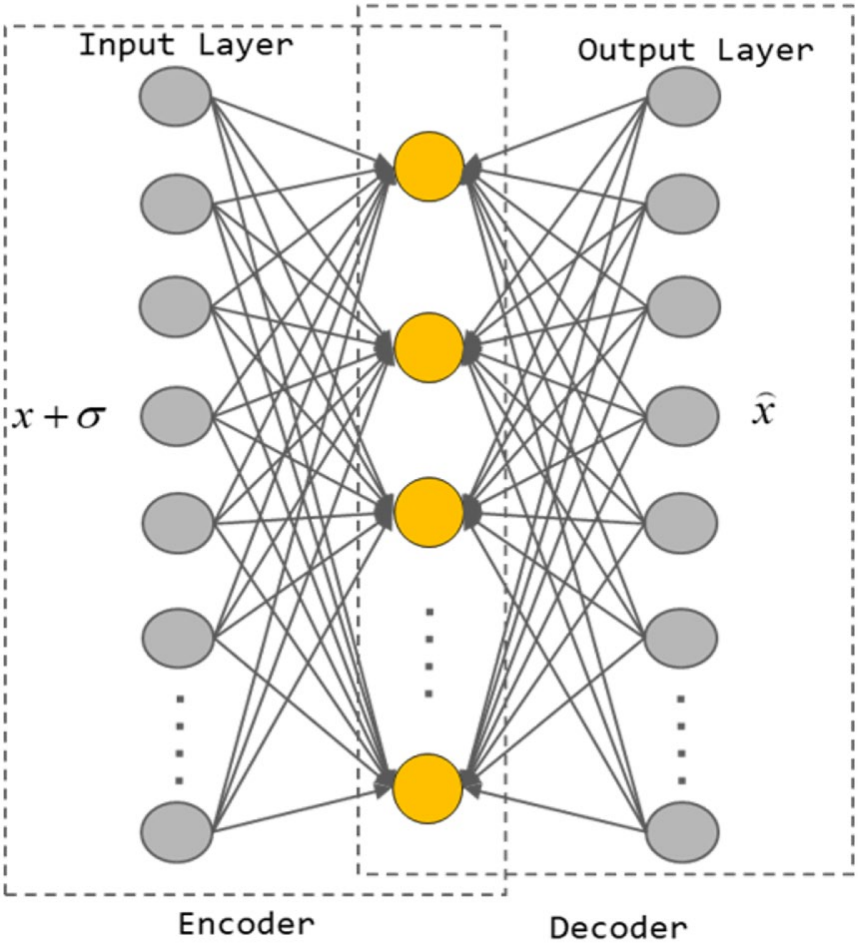
Hierarchical framework of DAE.

The network structure of DAE is shown in the Fig. 2.

SDAE stacks the DAEs into a deep network to obtain deep features of input data. The SDAE model destroys the original data *x* into ${\tilde{x}}$ by adding Gaussian noise $\sigma$, and then trains the SDAE model to get a better reconstructed signal.

### 1DCNN based on triplet loss

**Figure 3:**
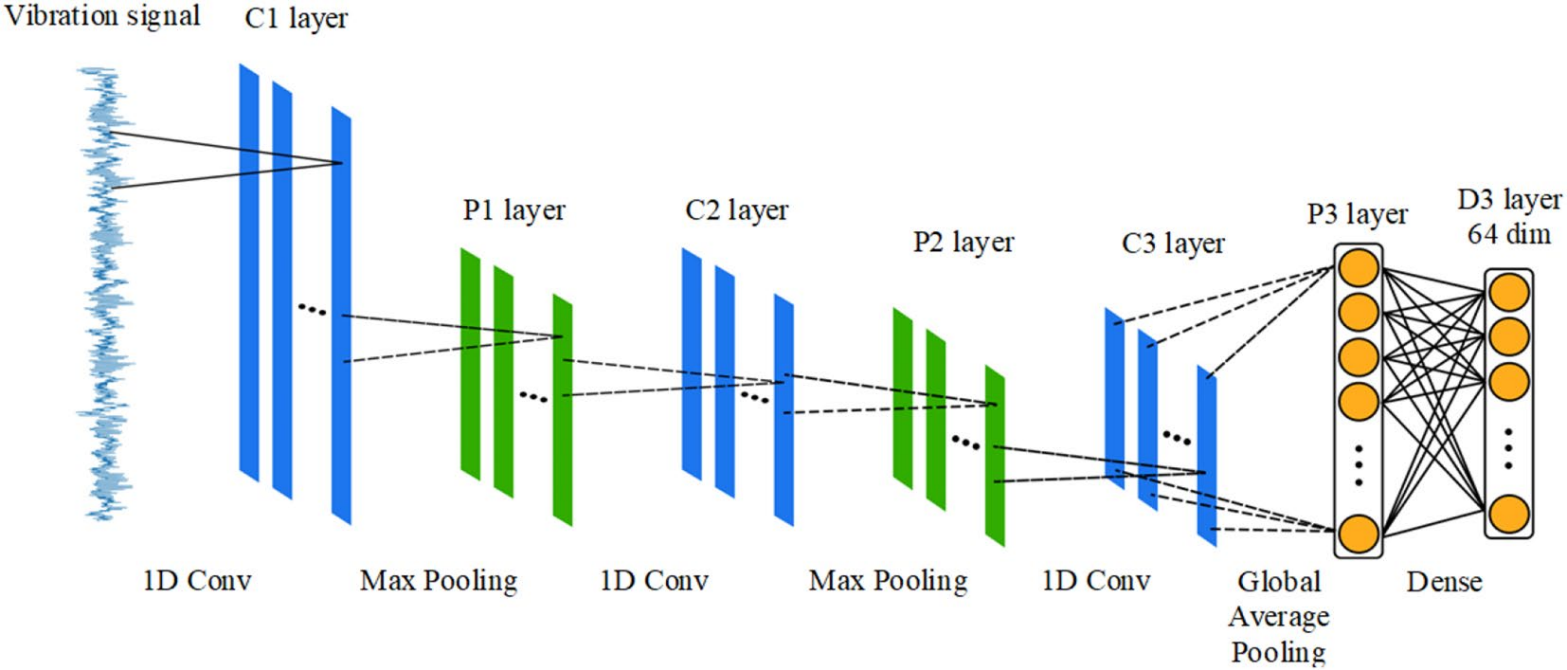
Hierarchical framework of 1DCNN.

We believe that in a small sample of bearing fault dataset, in order to extract the deep information in the waveform, the CNN model needs to have sufficient depth. The performance of end-to-end learning in small sample datasets is not stable enough, and it is difficult to learn a better distribution law. In order to better classify bearing faults, this paper proposes a SVM fault classification method using trplet network as data preprocessing. The triplet network converts the problem of the mapping relationship between learning data to classification into a problem of different relationships between learning data. This method has a good performance in small sample data. The established one-dimensional CNN model has 8 hidden layers, and the model structure is shown in Fig. 3.

Establish a triplet network based on the 1DCNN model. In order to train triplet network, the data is classified according to labels:

- *a*: anchor, any sample in the training set
- *p*: positive, a sample of the same category as *a*
- *n*: negative, a sample different from *a*

The triplet network output layer generally has fewer dimensions, which is equivalent to the high-order feature representation of the original signal. Input the same batch of data into the model, and calculate the triplet loss based on the sample label. the goal of triplet loss is to reduce The distance between *a* and *p*, and increases the distance between *a* and *n* in the embedding space. We need to introduce *margin* to make the distance between samples of different categories larger than *margin*, which can make the network encode samples of different categories farther. the loss of a triplet (*a*, *p*, *n*) is:

3 $$\begin{aligned} loss = \max \left( {\left\| {a - p} \right\| - \left\| {a - n} \right\| + margin,0} \right) \end{aligned}$$

**Figure 4:**
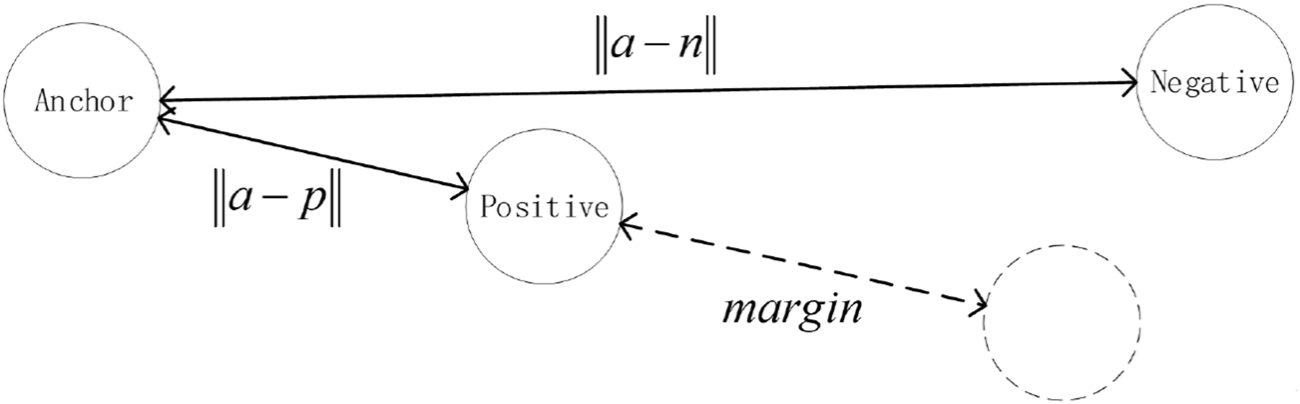
Easy triplets.

**Figure 5:**
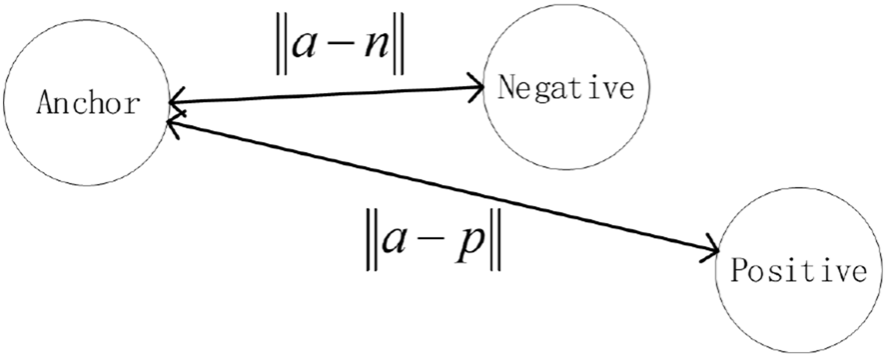
Hard triplets.

**Figure 6:**
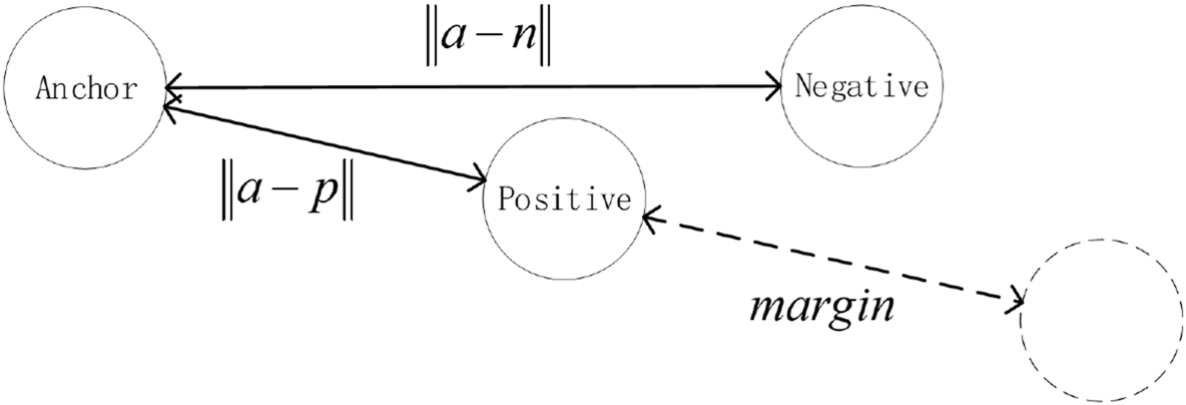
Semi-hard triplets.

In the formula, $\left\| {a - p} \right\|$ is the Euclidean distance between *a* sample and *p* sample, and $\left\| {a - n} \right\|$ is the Euclidean distance between *a* sample and *n* sample. The goal of the algorithm is to reduce the value of $\left\| {a - p} \right\|$ as much as possible to make the distance between *a* and *p* closer, and increase the value of $\left\| {a - n} \right\|$ as much as possible to make the distance between *a* and *n* longer. Make the value of $\left\| {a - n} \right\| - \left\| {a - p} \right\|$ small enough to achieve the goal of the algorithm. By calculating $\max \left( {\left\| {a - p} \right\| - \left\| {a - n} \right\| + m\arg in,0} \right)$ , we can control the aggregation degree of positive samples and the dispersion degree of negative samples in the feature space, so that the distance between positive samples and negative samples is greater than the margin value as much as possible. There are three situations when calculating triplet loss:

1. easy triplets: $loss=0$, $\left\| {a - p} \right\| + margin < \left\| {a - n} \right\|$. This situation does not need to be optimized. The distance between *a* and *p* is very close, and the distance between *a* and *n* is far and greater than the sum of the distance between *a* and *p* and the margin, as shown in the Fig. 4.
2. hard triplets: $\left\| {a - n} \right\| < \left\| {a - p} \right\|$, The distance between *a* and *p* is long, and this situation will produce a greater loss. As shown in Fig. 5.
3. semi-hard triplets: $\left\| {a - p} \right\|< \left\| {a - n} \right\| < \left\| {a - p} \right\| + margin$, The distance between *a* and *p* is short, and the distance between *a* and *n* is long, but the distance between *a* and *n* is less than the distance between *a* and *p* plus *margin*. This situation will produce less loss. As shown in Fig. 6.

Input all the training set data into the network to calculate the corresponding 64-dimensional embedding. Take one of the samples *a*. There are a total of *m* samples with the same label as sample *a*, forming set $P = \{ {p_1},{p_2},{p_3} \cdots {p_m}\}$, There are a total of *n* samples with labels different from sample *a*, forming set $N = \left\{ {{n_{\mathrm{{1}}}},{n_2},{n_3} \cdots {n_n}} \right\}$. Calculate the distance ${D_p}$ between *a* and all positive samples:

4 $$\begin{aligned} {D_p} = \left\{ {\left\| {a - {p_1}} \right\| ,\left\| {a - {p_2}} \right\| ,\left\| {a - {p_3}} \right\| , \ldots ,\left\| {a - {p_k}} \right\| , \ldots ,\left\| {a - {p_m}} \right\| } \right\} \end{aligned}$$

Calculate the distance between *a* and all negative samples to form set ${D_n}$:

5 $$\begin{aligned} {D_n} = \left\{ {\left\| {a - {n_1}} \right\| ,\left\| {a - {n_2}} \right\| ,\left\| {a - {n_3}} \right\| , \cdots ,\left\| {a - {n_s}} \right\| , \cdots ,\left\| {a - {n_n}} \right\| } \right\} \end{aligned}$$

Take one element in set ${D_p}$ and set ${D_n}$ respectively, and combine them with *a* to form a triplet. All possible combinations form set *T*:

6 $$\begin{aligned} T = \left\{ {\left. d \right| d = max\left( {\left\| {a - {p_k}} \right\| - \left\| {a - {n_s}} \right\| + margin,0} \right) } \right\} \end{aligned}$$

There are $m \times n$ combinations of elements in ${D_p}$ and ${D_n}$, the total number of samples is $m + n + 1$, and the value of *a* has $m + n + 1$ possibilities, so the size of the set *T* is $m \times n \times \left( {m + n + 1} \right)$. Select semi-hard triplets in set *T*, get set ${T_s} = \left\{ {{d_1},{d_2},{d_3} \cdots {d_a}} \right\}$ , and calculate triplet loss:

7 $$\begin{aligned} loss = \frac{1}{a}\sum \limits _{i = 1}^a {{d_i}} \end{aligned}$$

**Figure 7:**
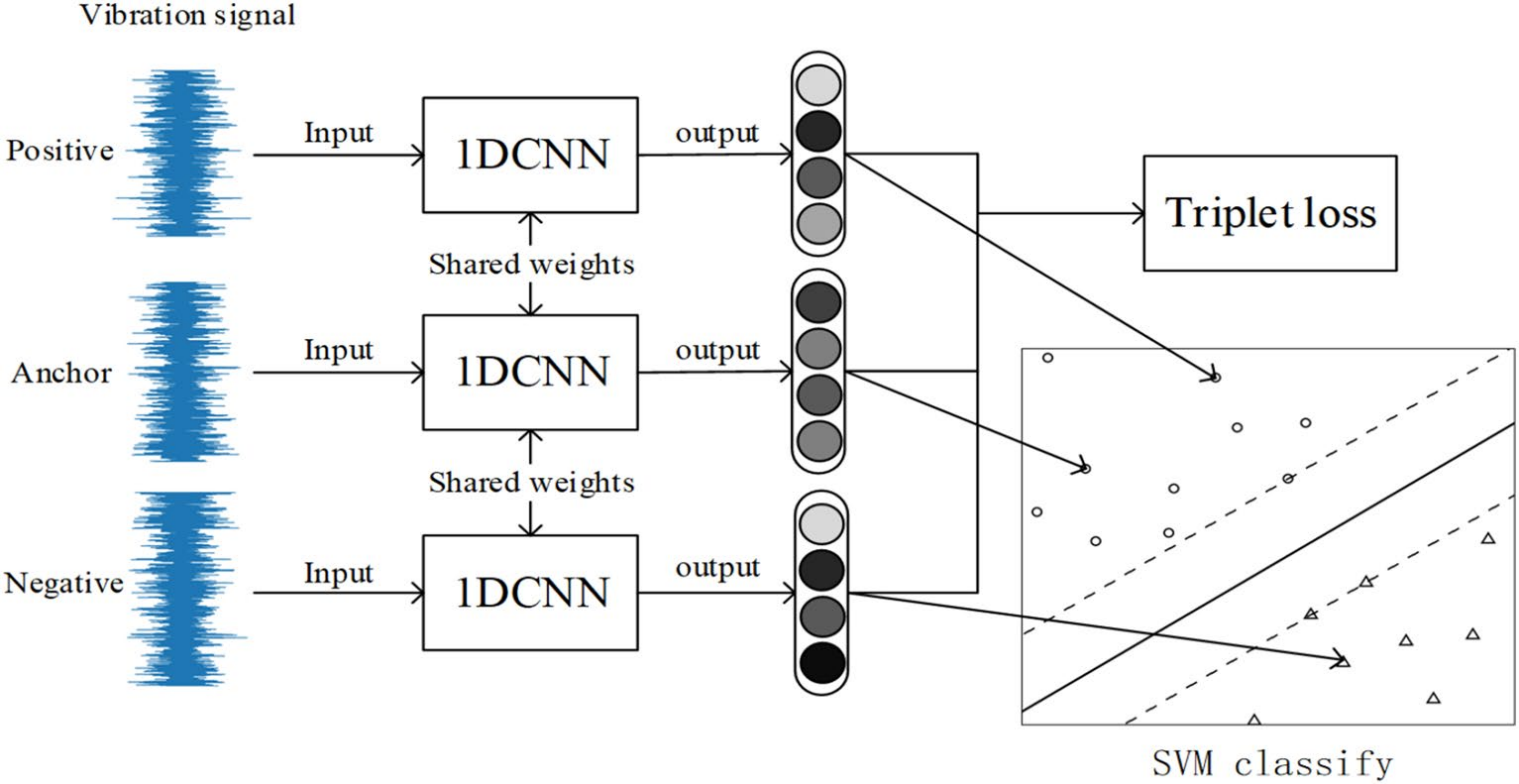
Training flow of triplet network and SVM.

**Figure 8:**
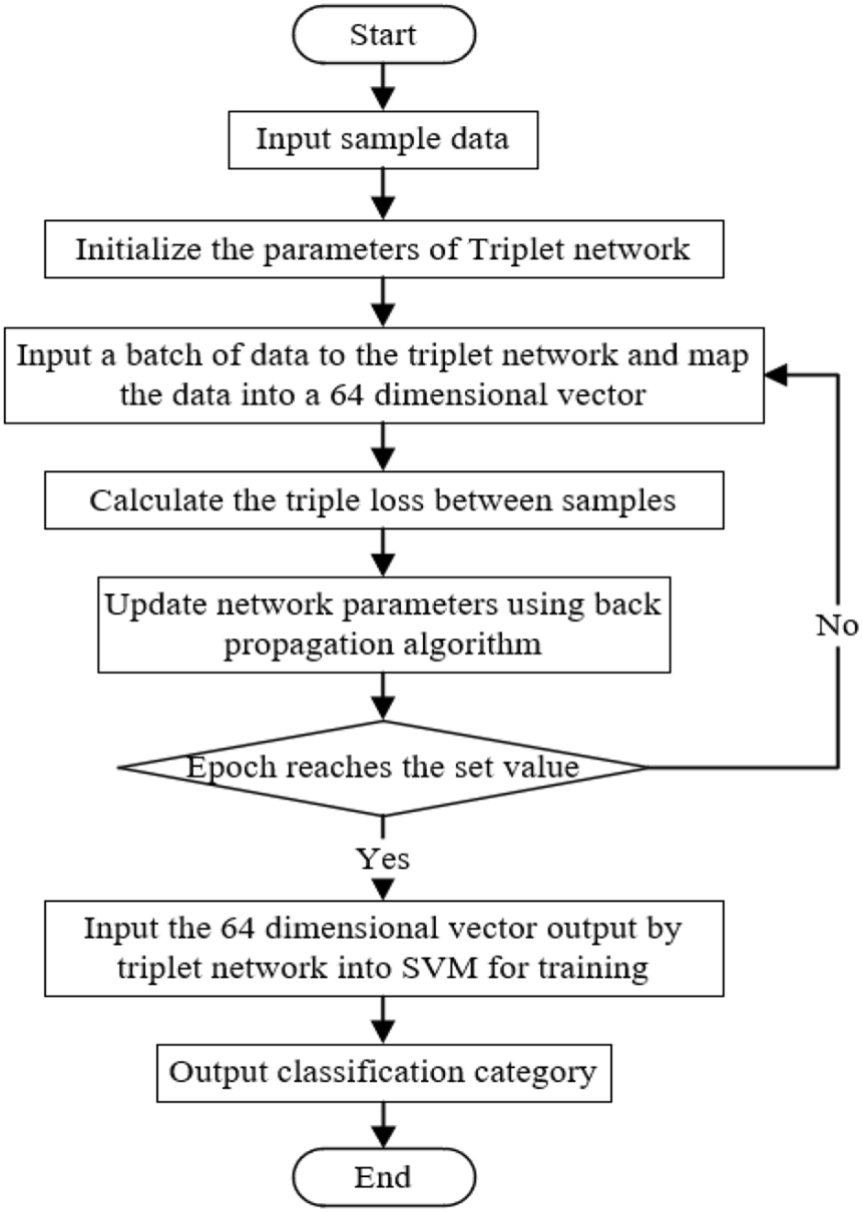
Block diagram of the proposed model.

Figure 7 shows the training process of triplet network and SVM. Figure 8 is block diagram of the proposed method. Firstly, input the labeled vibration data into the model. Then, initialize the triplet network model parameters, and input the collected bearing data set into triplrt network for training. The loss function of the model is defined as triplet loss, and the output of the model is a 64 dimensional vector. The trained model maps the samples to the high-dimensional feature space, and the distance between similar samples is small, and the distance between different samples is large. Finally, using high-dimensional features for SVM classification, we can judge whether the bearing fault and the fault location.

Different from AE, Triplet loss calculates the difference between the same sample and different samples, and backpropagates to update weights of the network so that the distance between the same samples is closer and the distance between different samples is longer.

## Validation of proposed method

### Data Castle bearings dataset

#### Dataset introduction

This paper uses the bearing data set provided by Data Castle to verify the method^38^. Data Castle collected the vibration information of normal and faulty bearings. There are three types of bearing faults, including inner race, outer race and ball. The bearings include three different diameters, so there are nine different bearing fault labels. The dataset we used is shown in Table 1.

**Table 1:** Data Castle bearings dataset.

Diameter	Fault location	Samples	Label
	Normal	177	0
Diameter1	Outer race	141	1
	Inner race	140	2
	Ball	52	3
Diameter2	Outer race	51	4
	Inner race	50	5
	Ball	47	6
Diameter3	Outer race	46	7
	Inner race	45	8
	Ball	43	9

**Figure 9:**
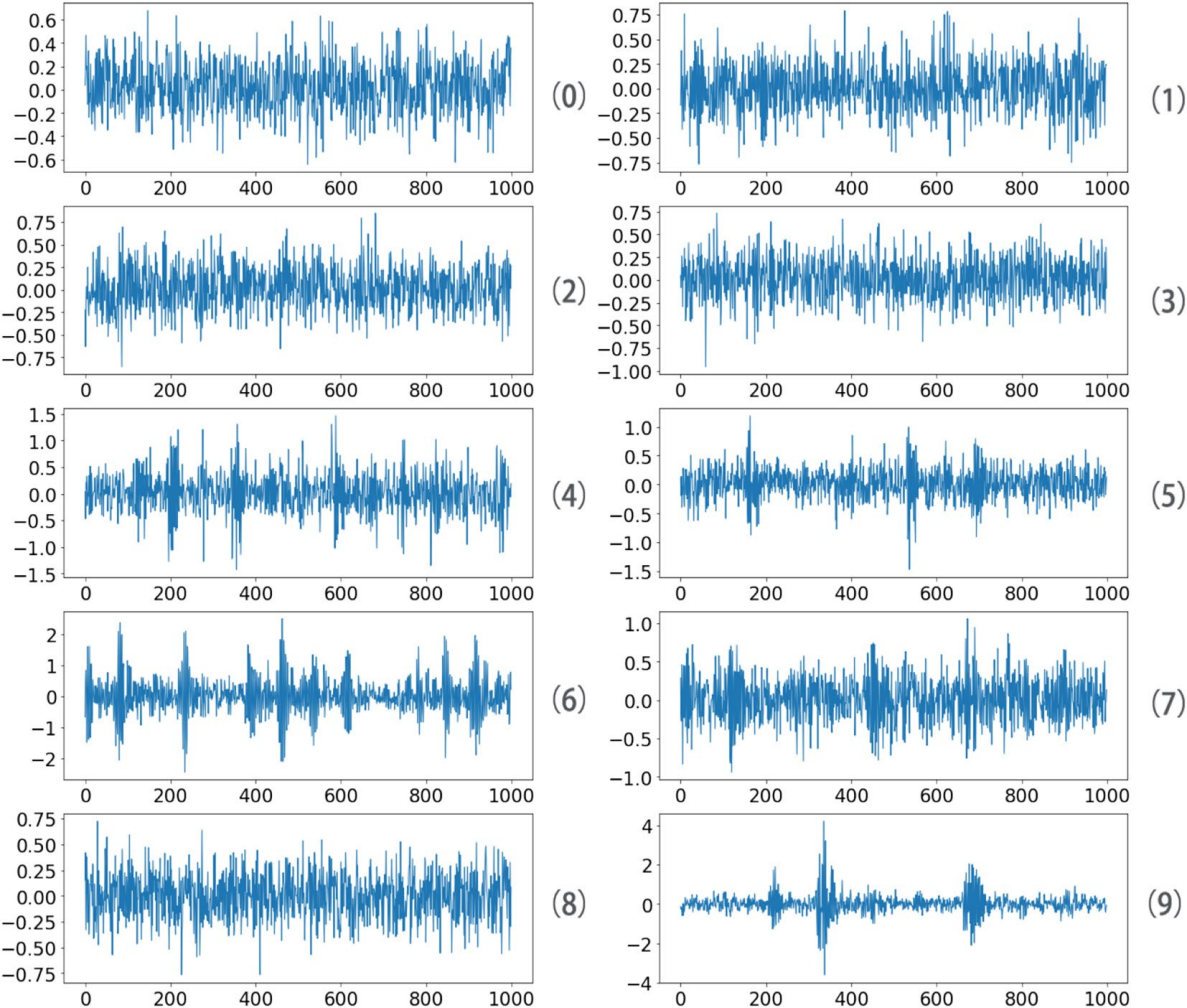
Fault bearing vibration data waveform.

In order to verify the robustness of the algorithm, we add white noise with mean value of 0 and standard deviation of 0.2 to the original data, and use the data after adding noise for model training to test whether the model can accurately judge the samples with insignificant features. The data after adding noise is shown in the Fig. 9.

After adding noise, the characteristics of data have become less obvious, and the similarity of similar characteristics decreases after adding random noise, which requires the model to have good robustness to deal with these changes.

#### Experiment

Experimental steps:

1. Process the vibration data of the bearing and add appropriate labels to the vibration data.
2. Add white noise to the vibration data to make the data characteristics less obvious.
3. Divided the collected raw vibration data into training dataset and test dataset, and trained the network.
4. Inputed all training samples into the model, calculated the high-order features of all training samples, and used the high-order features to train the SVM classifier.
5. Test samples are input into the model to train high-order features, and inputed the high-order features into the trained SVM classifier for classification to verify the effectiveness of the proposed method.

In order to verified feasibility and superiority of the method, standard SAE, SDAE, and CNN were used for comparison. All the following experiments were performed in python code.

- The key parameters of SAE: Encoder contains a 6-layer neural network, including three convolutional layers and three down-sampling layers. To map the original vibration data into a 64-dimensional space vector, decoder contains 3 deconvolution layers to restore the 64-dimensional space vector to vibration data. We use Gaussian kernel function, the penalty coefficient is 1, and the Gamma value is 1/64. The 64-dimensional space vector output by the Encoder is input to the SVM classifier for classification.
- The key parameter of SDAE: Gaussian noise is added to the original data. The mean value of the added Gaussian noise is 0, and the standard deviation is 0.05. Other parameters of SDAE are the same as SAE.
- The key parameters of the CNN model: The end-to-end CNN model has 6 hidden layers, the first layer is One-dimensional convolutional layer which has 32 convolution kernels, the second layer is down-sampling layer, the third layer is One-dimensional convolutional layer which has 64 convolution kernels. The fourth layer is down-sampling layer. The fifth layer is One-dimensional convolutional layer which has 128 convolution kernels. The sixth layer is global average pooling layer. The seventh layer is fully connected layer with 64 neurons. The output layer activation function is softmax. Optimizer is RMSprop. Learning efficiency is set to 0.0003, and the loss function uses sparse categorical crossentropy.
- The first layer of the Triplet network is convolutional layer with 32 convolution kernels. The second layer is downsampling layer. The third layer is convolutional layer with 64 convolution kernels. The fourth layer is downsampling layer. The fifth layer is convolutional layer with 128 convolution kernels. The sixth layer is global average pooling layer. The seventh layer is fully connected layer with 64 neurons. The eighth layer is L2 regular layer. Margin value is 1. Optimizer is RMSprop. The learning rate is 0.0003. The trained triplet network is equipped with feature mapping capabilities. SVM classifier are used for classification in the feature space. The kernel function of SVM is set to linear kernel, the penalty coefficient is 10.

Table 2 lists the average test accuracy of each model. Through analysis of the training results, it is found that the classification accuracy of triplet network+SVM reaches 96.31%, and the classification accuracy is much higher than SAE+SVM and DAE+SVM. Due to the lack of inter-sample reference, the traditional encoder network only pays attention to the difference between the decoded signal and the original signal, and may discard some subtle features. These features may have high weights when doing classification. The end-to-end CNN network is not as good as triplet network+SVM due to the small sample size.

**Table 2:** Test accuracy of each model.

Method	Dimension of input data	Diagnosis result
SAE+SVM	6000	86.50%
SDAE+SVM	6000	84.66%
CNN	6000	89.01%
Triplet network+SVM	6000	96.31%

**Figure 10:**
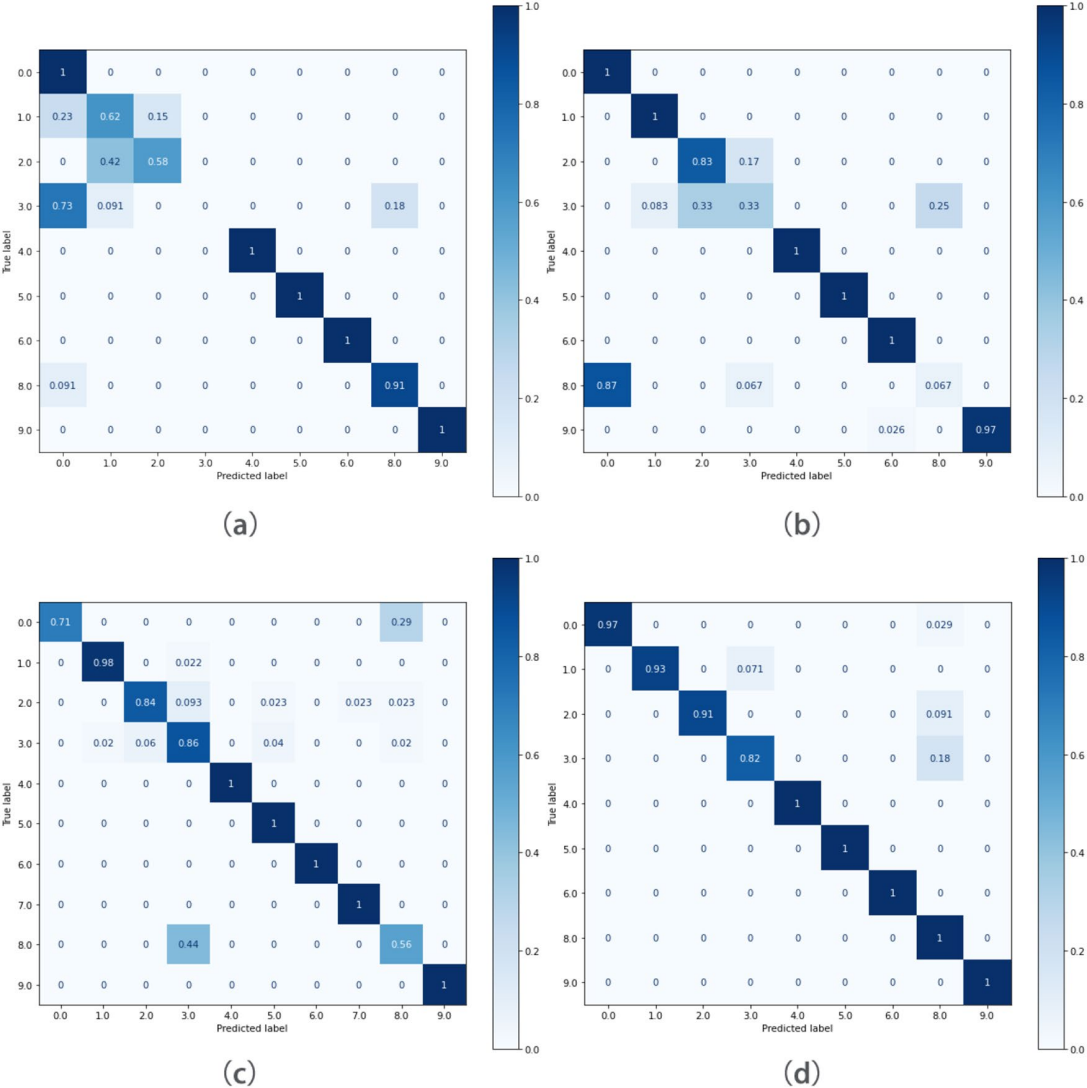
Multi-class confusion matrix of the proposed method: (**a**) SAE+SVM; (**b**) SDAE+SVM; (**c**) CNN; (**d**) Triplet network+SVM.

As shown in Fig. 10, it can be seen from the confusion matrix that the signals misclassified by SAE+SVM and DAE+SVM are similar to their correct classification, which means that the signal compressed by the autoencoder loses some information for classification. This causes the accuracy of the SVM classifier to decrease. the classification error of end-to-end CNN model reaches 89.01%. However, due to the insufficient sample size, it is difficult to further improve the predictive ability of the model. Training triplet network on a small sample dataset, the network learned how to distinguish samples with different labels. This makes the distance between samples of the same type smaller in the feature space, and samples of different types become larger in the feature space.

In order to compare the feature extraction capabilities of the methods, PCA dimensionality reduction was performed on the output of the three models. Select the first two principal components to visualize the features.

**Figure 11:**
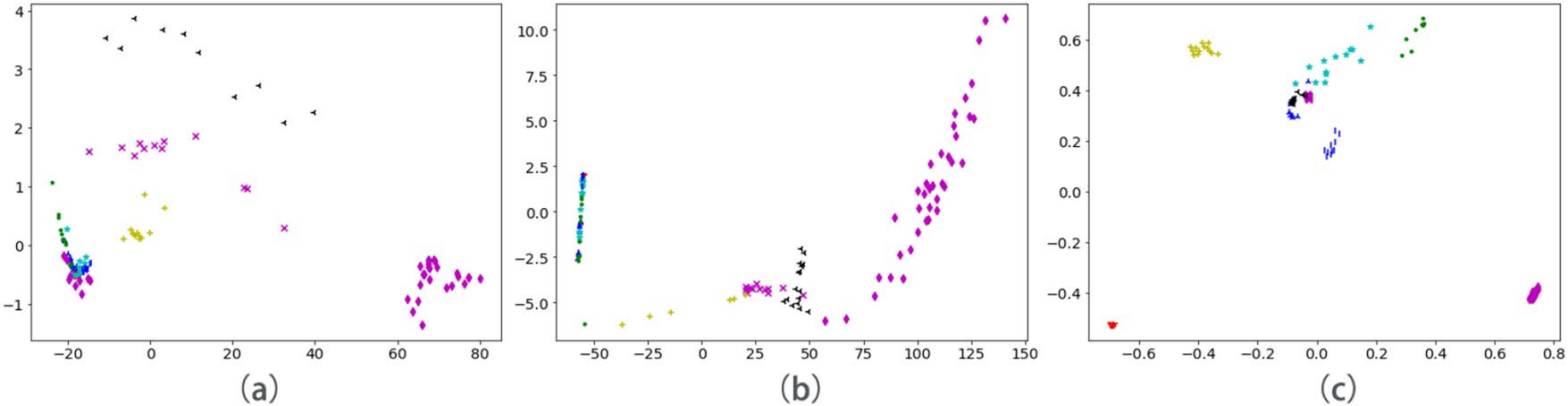
PCA dimension reduction feature visualization: (**a**) SAE; (**b**) SDAE; (**c**) Triplet network.

It can be seen from Fig. 11 that the different types of data that have been reduced by the Triplet network are far apart in the feature space, and the network has learned the differences between the data. However, the high-dimensional features learned by SAE and DAE cannot find the difference between different types of data well.

The Calinski-Harabaz Index(CH) is used to evaluate classification of feature spaces. The tightness within the class is measured by calculating the sum of the squares of the distance between each point in the class and the class center. The degree of separation of the dataset is measured by calculating the sum of squared distances from various center points. The function is:

8 $$\begin{aligned} CH(k) = {{\frac{{BGSS}}{{K - 1}}} \bigg {/} {\frac{{WGSS}}{{n - K}}}} \end{aligned}$$

*n* represents the number of samples in dataset, *K* represents the number of categories. The values of *WGSS* and *BGSS* are obtained by formula 9 and 10:

9 $$\begin{aligned} WGSS= & {} \frac{1}{2}\left[ ({n_1} - 1){\overline{d}} _1^2 + \cdots + ({n_k} - 1){\overline{d}} _k^2\right] \end{aligned}$$

10 $$\begin{aligned} BGSS= & {} \frac{1}{2}\left[ (K - 1){{\overline{d}} ^2} + (n - K){A_K}\right] \end{aligned}$$

11 $$\begin{aligned} {A_K}= & {} \frac{1}{{n - K}}\sum \limits _i^K {({n_i} - 1)({{{\overline{d}} }^2} - {\overline{d}} _i^2)} \end{aligned}$$

${\overline{d}} _j^2$ represents the average distance between samples of type *j*, $j = 1,2, \cdots ,k$. ${{\overline{d}} ^2}$ is the average distance between all samples.

The larger the CH index value, the smaller the intra cluster distance, the larger the intra cluster distance, the higher the degree of intra-class aggregation, the higher the degree of distinction between classes, and the easier it is to classify. The smaller the CH index value, the larger the distance within the class, and the smaller the distance between the classes, which makes it difficult to classify. The CH index values of SAE, SDAE, and triplet network are shown in Table 3.

**Table 3:** CH index of each algorithm.

Method	CH Index
SAE	36.42
SDAE	73.74
Triplet network	1015.91

### XJTU-SY bearing dataset

#### Introduce

**Figure 12:**
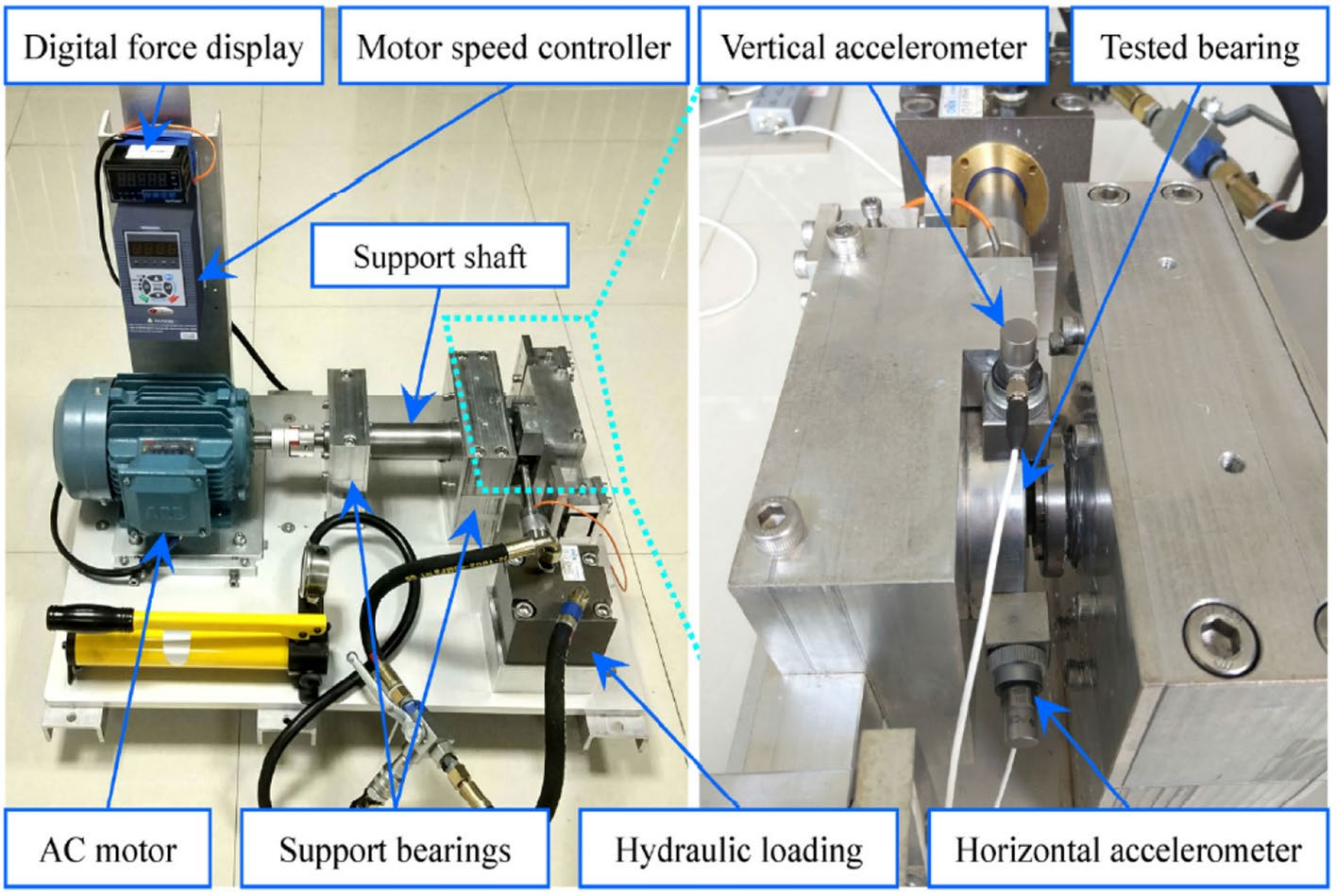
Testbed of rolling element bearings.

The bearing testbed consists of supporting shaft, motor governor, AC induction motor, hydraulic loading system and two supporting bearings. The testbed is designed for accelerated degradation test of bearings under different working condition. The radial force is exerted on the shell of the tested bearing by the hydraulic loading system, and the speed controller of AC induction motor sets and maintains the speed of the whole shaft system^39^. As shown in Fig. 12.

The acceleration sensor used in the experiment is PCB 352c33. The sensor is installed on the magnetic base, which is fixed on the vertical and horizontal direction of the test bearing. The dynamic signal collector uses DT 9837. the sampling frequency of the original data is 25.6 khz, the sampling interval of the original data is 1 min, and the sampling time of the original data is 1.28 s. Table 4 shows the detailed information of each tested bearing, including its corresponding operating conditions, bearing lifetime and failure location.

**Table 4:** XJTU-SY bearing dataset.

Operating condition	Dataset	Lifetime	Fault location	Label
Condition 1(35Hz/12kN)	Bearing1	2h3min	Outer race	1000
	Bearing2	2h41min	Outer race	1000
	Bearing3	2h38min	Outer race	1000
	Bearing4	2h2min	Cage	0100
	Bearing5	52min	outer race and Inner race	1010
Condition 2(37.5Hz/11kN)	Bearing6	8h11min	Inner race	0010
	Bearing7	2h41min	Outer race	1000
	Bearing8	8h53min	Cage	0001
	Bearing9	42min	Outer race	1000
	Bearing10	5h39min	Outer race	1000
Condition 3(40Hz/10kN)	Bearing11	42h18min	Outer race	1000
	Bearing12	41h36min	Outer race, ball, cage and Inner race	1111
	Bearing13	6h11min	Inner race	0010
	Bearing14	25h15min	Inner race	0010
	Bearing15	1h54min	Outer race	1000

**Figure 13:**
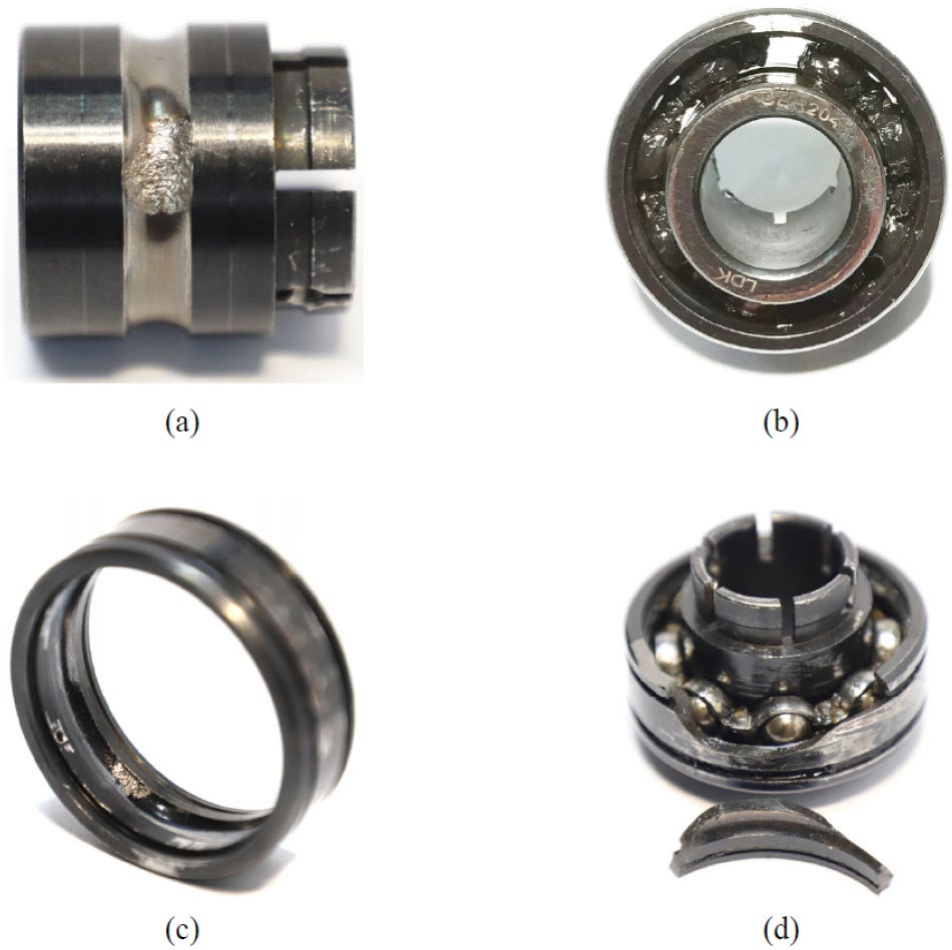
Failure bearings: (**a**) inner race wear; (**b**) cage fracture; (**c**) outer race wear; (**d**) outer race fracture.

Figure 13 shows the failure bearings. It can be observed that the bearing failure is mainly caused by outer race wear, inner race wear, outer race fracture and cage fracture. More than two types of composite damage may occur to the bearing. Compound damage has multiple waveform characteristics of damage locations, which increases the difficulty of classification. The method adopted in this paper is to assign separate labels for compound damage.

#### Experiment

XJTU-SY Bearing Dataset contains the full lifecycle vibration signals of 15 bearings. In order to divide the dataset, we take the data with amplitude less than 3g as the vibration data of normal bearing and the data greater than 5g as the vibration data of faulty bearing. Since the collected signals are vibration signals in both the vertical and horizontal directions, the two channels of the vertical vibration signal and the horizontal vibration signal are used as the input of the model.

**Figure 14:**
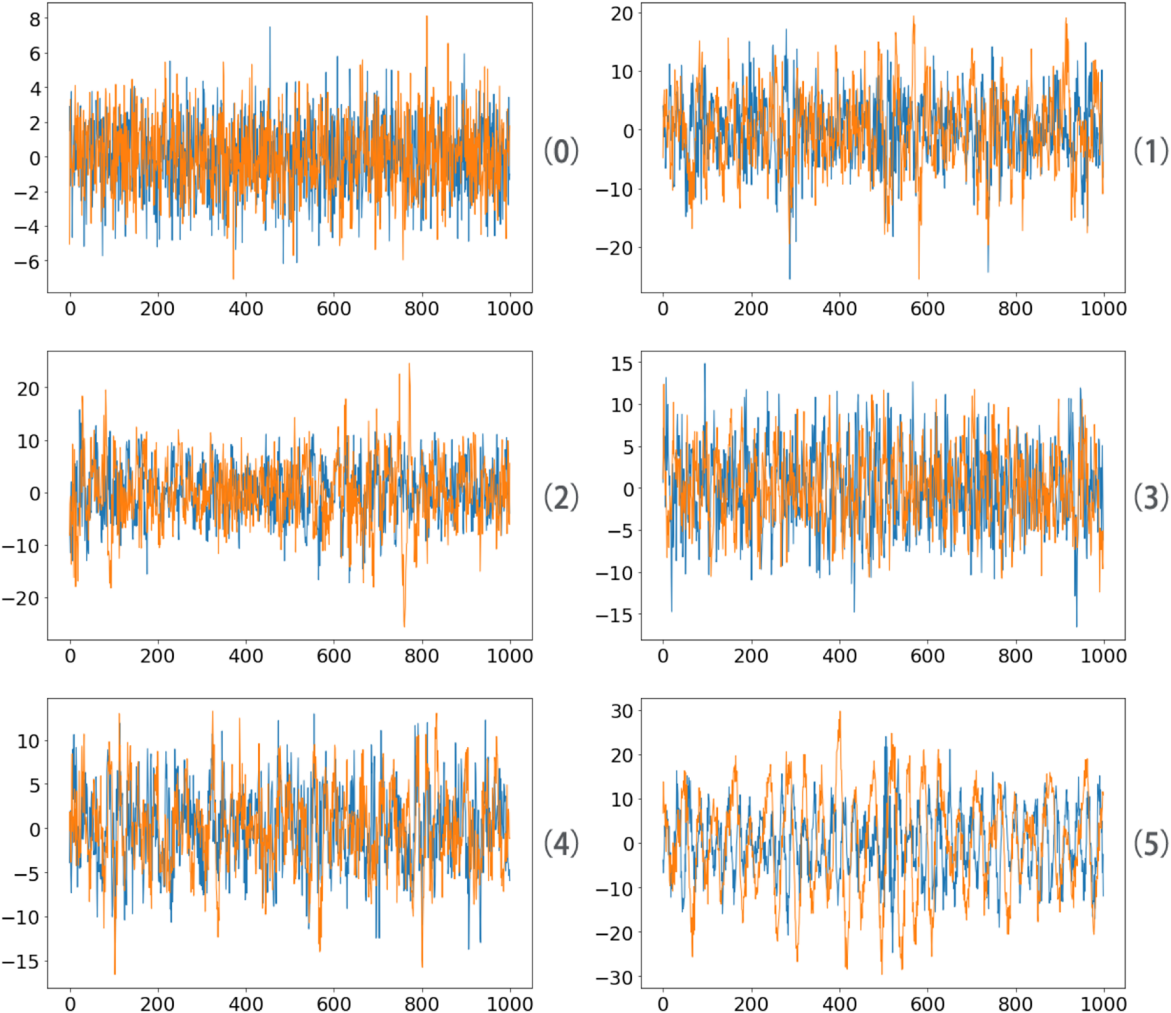
Fault bearing vibration data waveform.

In order to verify the robustness of the four models, we add white noise with mean value of 0 and standard deviation of 2 to the original data, as shown in the Fig. 14, in which the blue curve is horizontal vibration data and the Yellow curve is vertical vibration data.

Train SAE, SDAE, CNN and triplet network models, and input test dataset into the model. The results are shown in Table 5.

**Table 5:** Diagnosis result of each algorithm.

Method	Dimention of input data	Diagnosis result
SAE+SVM	2000*2	88.49%
SDAE+SVM	2000*2	89.41%
CNN	2000*2	86.72%
Triplet network+SVM	2000*2	97.09%

It can be seen that the results of triplet network+SVM on XJTU-SY Bearing Dataset are better than CNN, DAE+SVM and SAE+SVM. The classification accuracy of the triplet network+SVM reached 97.09%. The self-encoder is better than the end-to-end CNN.

## Conclusion

We propose a bearing faults classification method based on triplet network and SVM. In this paper, the proposed method consists of two main steps. First, we propose a one-dimensional convolution model based on Triplet loss. The input of the model is original vibration data of bearings and the output is 64-dimensional high-order features. The loss function calculates the L2 norm distance between samples in the high-order feature space. this makes the distance between the same category closer, the distance between different category farther. Second, use SVM to classify in the high-order feature space. Two examples were given to illustrate the superiority of this method in dealing with small sample classification problems. The triplet network based on the CNN model can well extract the high-order features of the one-dimensional vibration signal, and these features can well express the difference between the signals. Compared with CNN, DAE+SVM, SAE+SVM, the algorithm performs better on small sample classification problems, and can accurately determine the fault category of bearing.

Although the existing algorithms have conducted in-depth research on the fault classification of rotating machinery, there is still a lack of systematic research on the early warning of fault. Next, we will study the early fault diagnosis and fault location identification of bearing, so as to predict the possible faults of bearing earlier. Early warning can avoid equipment damage caused by bearing failure.

For data citations of datasets uploaded to e.g. *figshare*, please use the howpublished option in the bib entry to specify the platform and the link, as in the Hao:gidmaps:2014 example in the sample bibliography file.
